# The role of parental identity in experiencing climate change anxiety and pro-environmental behaviors

**DOI:** 10.3389/fpsyg.2025.1579893

**Published:** 2025-03-25

**Authors:** Mariana Pinho

**Affiliations:** ECOMARE, Centre for Environmental and Marine Studies, Department of Biology, University of Aveiro, Aveiro, Portugal

**Keywords:** climate change anxiety, pro-environmental behavior, parental identity, environmental identity, parenting

## Abstract

**Introduction:**

Climate change is one of society's most severe crisis, presenting a health threat to humans with serious impacts on mental health. Climate anxiety has been identified as an important mental health consequence of climate change.

**Methods:**

The current study examined the role of social psychological characteristics on climate change anxiety and pro-environmental behavior, using a nationally representative sample of Portuguese parents who completed extensive questionnaires.

**Results:**

More central parental identities negatively correlated with and predicted climate change anxiety, revealing that a central parental identity can be a protective factor against mental health issues. Parental identity centrality also predicted greater engagement in pro-environmental behavior. The findings further showed that environmental identity and climate change perceptions were positively related and predicted higher levels climate change anxiety and pro-environmental behavior. Finally, parental identity centrality was linked to greater pro-environmental behavior through climate change anxiety, bringing important contributions to research on the underlying mechanisms that shape pro-environmental behavior.

**Discussion:**

The findings shed light on the complex mechanisms underlying and influencing climate anxiety and pro-environmental behavior, necessary to mitigate the acute consequences of the climate crisis.

## 1 Introduction

Climate change is one of society's most serious threats of the 21^st^ century. It presents a health threat to humans with severe impacts on mental health, not limiting it to just an environmental issue (e.g., Clayton and Karzsia, 2020; Clayton and Manning, 2018; Dodds, 2021; Hickman et al., 2021). Recent studies have explored the connection between climate change and mental health through emotional responses such as increased anxiety (e.g., Clayton and Karzsia, 2020; Dodds, 2021). Climate anxiety can be defined as “heightened emotional, mental or somatic distress in response to dangerous changes in the climate system” (Dodds, 2021, p. 222). Despite the implications of climate anxiety being maladaptive, researchers have argued that experiencing anxiety can be an appropriate response to the gravity of climate change (Clayton and Karzsia, 2020) and related to pro-environmental behavior (Chapman and Peters, 2024; Whitmarsh et al., 2022). However, climate anxiety has been associated with poor mental health and functional impairment, such as depression, anxiety, stress and insomnia (Boluda-Verdú et al., 2022; Patrick et al., 2023; Schwartz et al., 2023; Sciberras and Fernando, 2022). Those effects occur even amongst individuals who are not directly impacted by climate change (e.g., Lawrance et al., 2022; Manning and Clayton, 2018; Ogunbode et al., 2022). As a growing proportion of the population experiences the acute effects of climate change, they are also likely to increasingly suffer from climate anxiety. While a rising body of research has attempted to understand and explain the impact of climate change on mental health, it has mainly focused on the prevalence and demographic determinants of climate change anxiety (e.g., Clayton and Karzsia, 2020; Patrick et al., 2023; Schwartz et al., 2023). Consequently, much less is known about the operation of social psychological mechanisms in climate change anxiety and pro-environmental behavior.

The current study aims to explore the role of social psychological characteristics, namely parental identity centrality, environmental identity and climate change perceptions in climate change anxiety and pro-environmental behavior. To examine the links with social psychological characteristics and its underlying processes, an original, nationally representative survey of Portuguese parents was conducted. Social and cultural environment shape one's identity and in turn, guide and motive behavior (Stryker and Burke, 2000; Yarrison, 2022). It is therefore vital to understand the complex mechanisms underlying and influencing climate anxiety and pro-environmental behavior to mitigate the severe consequences of the climate crisis.

### 1.1 Climate anxiety impact in children and youth

Climate change is a pressing social issue with enormous influence on the lives of future generations (Clayton, 2020; Otto et al., 2019). Globally younger generations express high levels of worry regarding climate and Portuguese children and youth are no exception (Hickman et al., 2021). Previous research has emphasized how higher exposure to climate change and greater physical vulnerability, makes children and adolescents more susceptible to the health impacts of climate change, including climate change anxiety (e.g., Burke et al., 2018; Hickman, 2024; Sheffield and Landrigan, 2011; UNICEF, 2021). For a full review of the factors that influence climate anxiety in children and adolescents see Crandon et al. (2022) and Léger-Goodes et al. (2022).

Family (parents in particular) appears to have an impact on children's awareness and response to climate change (e.g., Léger-Goodes et al., 2022; MacKay et al., 2020). For example through their values, behaviors and communication, as the way parents share, discuss and react to climate change influence the way children respond to it (e.g., Gong et al., 2021; Iwaniec and Curdt-Christiansen, 2020). The opposite is also true, as children influence their parents concerns regarding climate (Lawson et al., 2019). Therefore, as parents are usually the primary agents of socialization, understanding the association of their social psychological characteristics on climate anxiety and pro-environmental behavior can provide insight into how they can be better role-models for their children.

### 1.2 Identity theory

Identity theory (Stryker, 1980, 2008) explains behavior in terms of the self and society, proposing that different expectations are associated with different social roles. According to the theory, identities are internalized role expectations together with the meanings that an individual attaches to them (Stryker, 1980; Stryker and Burke, 2000). The theory suggests that individuals have multiple identities organized in a hierarchy of importance (Merolla et al., 2012; Stryker, 1980, 2008). Identity centrality refers to the importance one consciously attributes to an identity, meaning how central certain parts of the self are (Rosenberg, 1979; Stryker and Serpe, 1994). Identity theory assumes that more central identities guide behavior to a greater extent than less central ones, predicting that more time and effort will be dedicated to enactment of an identity with higher centrality (Stryker and Burke, 2000; Stryker and Serpe, 1994). For example, previous studies have demonstrated that individuals with more central identities have higher involvement in childcare and lower involvement in paid work (Gaunt and Scott, 2014; Gaunt et al., 2024; Pinho and Gaunt, 2023).

### 1.3 Parental identity and mental health

As much as parenthood can be rewarding and give meaning to one's life (e.g., Musick et al., 2016; Nomaguchi and Milkie, 2020), it can also bring psychological challenges as it demands adaption and impacts one's sense of identity (Lawrence et al., 2008; Piotrowski et al., 2023; Sanders et al., 2022). According to developmental theories, stable and mature identities are a source of resilience and wellbeing (Erikson, 1968). In the case of parental identity, research has shown it plays an important role in parents' physical and mental health (e.g., Schrooyen et al., 2021; Fadjukoff et al., 2016; Piotrowski, 2018; Wickrama et al., 1995). Specifically, when parents have a clear and coherent parental identity, they experience higher levels of mental health (i.e. lower depression and anxiety), better parental adaptation, more positive parental experiences and higher life satisfaction (Fadjukoff et al., 2016; Gyberg and Frisén, 2017; Jungert et al., 2015; Piotrowski, 2018; Piotrowski et al., 2024; Schrooyen et al., 2021).

On the other hand, when doubts about whether becoming a parent was a good decision, low identification with the parental role and parental identity diffusion occur, parents are more likely to experience mental health issues, such as anxiety, depression, stress, internalizing problems and parental burnout (Fadjukoff et al., 2016; Meca et al., 2020; Mikolajczak et al., 2019; Piotrowski, 2018, 2021, 2023; Piotrowski et al., 2024; Schrooyen et al., 2021). Therefore, a well-formed, central parental identity appears to be a protective factor against mental health difficulties.

### 1.4 Climate change anxiety, social psychological factors and pro-environmental behavior

Climate change anxiety is the distress related to concerns and perceptions about the impact of climate change (Clayton, 2020). Previous research identified socio-demographic characteristics that predict higher levels of climate anxiety. Namely, being younger, a woman, having left-wing ideologies, and direct experience of climate change (Asgarizadeh et al., 2023; Clayton and Karzsia, 2020; Hickman et al., 2021; Verplanken et al., 2020; Whitmarsh et al., 2022; Wullenkord et al., 2021). Research has also explored the role of environmental values and identity on climate anxiety (e.g., Clayton and Karzsia, 2020; Galway et al., 2021).

Environmental identity can be defined as the personal association to a part of the non-human natural environment (Clayton, 2003, 2012). It has been positively associated with pro-environmental behaviors, greater attention to environmental issues and efforts to prevent climate change (e.g., Ajibade and Boateng, 2021; Brick and Lai, 2018; Mackay and Schmitt, 2019; Whitburn et al., 2020), as well as a reliable predictor of pro-environmental behavior (e.g., Brügger et al., 2021; Tam, 2013; Young et al., 2020).

The relationship between environmental identity and climate change perceptions has also been explored. Research has shown that environmental identity negatively predicts climate change denial (Nartova-Bochaver et al., 2022; Ucar et al., 2023). Research has identified three types of climate change perceptions: the degree to which one perceives climate change as real, caused by humans and as having negative consequences. Generally, climate change is believed to be real, to have anthropogenic origins and negative consequences worldwide (Leiserowitz et al., 2021; Steg, 2018, 2023).

Climate change perceptions are related to pro-environmental behavior and support for mitigation policies (Brink and Wamsler, 2019; Ding et al., 2011; Hornsey et al., 2016; Steg, 2023; van Valkengoed et al., 2021), being also a predictor of pro-environmental behavior depending on how it is framed (Bain et al., 2012). Additionally, stronger climate change perceptions have been associated with greater intentions to adopt adaptation behaviors (van Valkengoed et al., 2024).

### 1.5 Present study

Despite a growing body of research that has attempted to understand and explain climate anxiety and pro-environmental behavior (e.g., Clayton and Karzsia, 2020; van Valkengoed et al., 2024), the operation of social psychological mechanisms in climate change anxiety and pro-environmental behavior remains largely unexplored. Therefore, the present study aims to deepen our understanding of the unique contributions of social psychological characteristics to climate anxiety and pro-environmental behavior. To this end, a large, representative sample of Portuguese parents was recruited, and the following questions were explored:

- *Research Question 1*: What socio psychological characteristics associate with and predict climate anxiety?
- *Research Question 2*: What socio psychological characteristics relate and influence pro-environmental behavior?

These questions were explored in a sample of parents living in Portugal. Portugal comprises three geographical areas, namely mainland in the European Continent and two archipelagos in the Atlantic Ocean (Azores and Madeira). The country has experienced numerous effects of climate change, from heat waves, flooding to droughts (Cardoso et al., 2019; Carvalho, 2024; Turco et al., 2019). Due to its extensive and highly populated coastline Portugal also faces a significant threat from sea level rise. Over the past few years, extreme wildfires have also been more frequent and intense in Portugal (Carvalho, 2024; Turco et al., 2019). Despite efforts to implement mitigation and adaption measures, Portugal remains one of the European countries with greatest vulnerability to climate change (Cardoso et al., 2019; Carvalho, 2024; Climate Change Performance Index, 2025).

## 2 Method

### 2.1 Participants

A total sample of 2,055 adults (1,035 men and 1,020 women) met the criteria of being Portuguese residents who had at least one child. Participants' socio-demographic characteristics can be found in Table 1. Participants' ages ranged from 18 to 74 years old (*M* = 51.23, *SD* = 11.77), more than half had a university degree (51.8%) and lived in a city (64.8%). The age of the youngest child ranged from 2 months to 56 years (*M* = 18.64, *SD* = 12.14).

**Table 1:** The demographic characteristics of the participants.

Gender	
Male	50.4%
Female	49.6%
Age	
18–34	9.8%
35–54	43.3%
55–74	46.9%
Education	
Less than high school/secondary education	10.1%
High school/secondary education diploma	32.3%
Post-secondary non-higher education	5.8%
Higher education/academic degree	51.8%
Marital/relationship status	
Single	6.2%
Married/civil partnership	76.5%
Widower	2%
Divorced/separated	15.3%
Monthly household income	
≤ €590	4.1%
€590– €891	9.5%
€891–€1,688	28.6%
€1,688–€2,083	16.9%
€2,083–€3,071	24.1%
€3,071–€6,720	15.4%
≥€6,720	1.4%
Professional status	
Student	0.7%
Employed	73.1%
Retired	16.4%
Unemployed	6%
Homemaker	2.8%
Other	1%
Number of children	
1	45.8%
2	42.5%
3–6	11.7%

### 2.2 Measures

#### 2.2.1 Identity centrality

The psychological centrality of participants' identities was assessed using Gaunt and Scott's (2014) measure. Participants were presented with a list of eight identities (friend, sibling, wife/husband/partner, work, son/daughter, parent, national identity, religious identity) and the option to add other identities to the list was also given (for similar lists, see Pinho and Gaunt, 2023). Participants were asked to distribute 100% between various identities, in a way that reflected the extent to which each identity was important to them. This measure allowed participants to express the equal importance of two or more identities by allocating them equal percentages. The percentages allocated to parental identities were then coded to obtain participants' psychological centrality scores.

#### 2.2.2 Climate change anxiety

Climate anxiety as a psychological response to climate change was measured using Clayton and Karzsia's (2020) 13-item instrument. The measure is composed of two subscales: (a) cognitive impairment (e.g., “*Thinking about climate change makes it difficult for me to sleep*”) and (b) functional impairment (e.g., “*I have problems balancing my concerns about sustainability with the needs of my family*”). Responses were indicated on a 5-point scale ranging from 1 = *Never* to 5 = *Almost always*. The respondent's average score on each dimension was computed. Cronbach's alphas for these dimensions were 0.91 and 0.89, respectively. The average of all 13 items was also calculated to obtain a total climate change anxiety score. Cronbach's alpha for the overall climate change anxiety scale was 0.94.

#### 2.2.3 Environmental identity

Participants' environmental identity was assessed using Clayton et al.'s (2021) scale. The measure included 14 items that measure individual differences in a stable sense of interdependence and connectedness with nature. Responses were indicated on a scale from 1 = *Not at all true of me* to 7 = *Completely true of me*. The average score was computed to measure participants' overall environmental identity. Cronbach's alpha for this measure was 0.93.

#### 2.2.4 Pro-environmental behavior

Conservation behaviors with the greatest impact on the environment were measured using Markle's (2013) scale. The scale included six pro-environmental behaviors where responses were indicated on a scale from 1 = *Never* to 5 = *Always*. The average score was computed to measure participants' conservation behaviors. Cronbach's alpha for this measure was 0.71.

#### 2.2.5 Climate change perceptions

Participants' perceptions of climate change were assessed using van Valkengoed et al.'s (2021) scale, which included three types of perceptions: (a) reality (e.g., “*I believe that climate change is real*”); (b) causes (e.g., “*Human activities are a major cause of climate change*”); and (c) valence of consequences (e.g., “*Climate change will bring about serious negative consequences*”). Participants used a 7-point Likert-scale from 1 = *Strongly disagree* to 7 = *Strongly agree*. An average of all climate change perception items was also calculated to create a total score. Cronbach's alpha for this measure was 0.96.

#### 2.2.6 Socio-demographic variables

Participants indicated their age, gender, occupation, level of education and marital status. Participants also reported the age of their youngest child, the total number of children, their individual monthly income on a seven-point scale ranging from 1 (< €*590*) to 7 (>€*6,720*).

### 2.3 Procedure

Participants were recruited using an online questionnaire administered to members of the Qdata panel of 860,000 individuals. Qdata is one of the leading survey companies in Portugal and has a panel of nationally representative members. The sample size for the study was calculated with a margin of error of 2% and confidence level of 95% of the total Portuguese current population (10,639,726). Stratified random sampling was applied and the sample contained individuals from all demographic groups in the same proportions as the whole Portuguese population. Emails were sent to panelists selected at random from the base sample of the required profile. The responding sample was weighted to the profile of the sample definition to provide a representative reporting sample. Participation in the study was voluntary and anonymous. The completion of the questionnaire took 15 min on average. Afterwards, participants were thanked and debriefed.

## 3 Results

### 3.1 Preliminary analysis

Means, standard deviations, and Pearson correlations among the three socio psychological measures, number of children, age of the youngest child, climate change anxiety and pro-environmental behavior are presented in Table 2. Consistent with previous findings (e.g., Galway et al., 2021), climate change anxiety was positively related with environmental identity (*r* = 0.24) and climate change perceptions (*r* = 0.10).

**Table 2:** Means, standard deviations, and correlations among study measures.

**Variables**	**1**	**2**	**3**	**4**	**5**	**6**	**7**
1. Climate change anxiety	–						
2. Pro-environmental behavior	0.08^***^	–					
3. Parental identity centrality	–0.13^***^	0.07^**^	–				
4. Environmental identity	0.24^***^	0.36^***^	–0.01	–			
5. Climate change perceptions	0.10^***^	0.25^***^	0.08^***^	0.32^***^	–		
6. Number of children	0.01	0.01	0.01	0.06^*^	–0.01	–	
7. Age of youngest child	–0.04	0.10^***^	–0.04	0.01	0.01	0.02	–
M	1.76	4.25	28.55	5.71	6.12	1.69	18.64
SD	0.67	0.57	16.55	0.88	1.00	0.78	12.14

Higher scores on all measures reflect higher levels of the construct. ^*^p < 0.05; ^**^p < 0.01; ^***^p < 0.001.

Table 2 also shows that parental identity centrality was negatively related with climate change anxiety (*r* = −0.13) and positively related with pro-environmental behavior (*r* = 0.07).

Echoing previous research, pro-environmental behavior was positively related with climate change anxiety (*r* = 0.08) (e.g., Hogg et al., 2021; Verplanken et al., 2020; Wullenkord et al., 2021), environmental identity (*r* = 0.36) and climate change perceptions (*r* = 0.25) (e.g., Ajibade and Boateng, 2021; van Valkengoed et al., 2021, 2022; Whitburn et al., 2020). Results in Table 2 further demonstrate that pro-environmental behavior was positively correlated with age of youngest child (*r* = 0.10). Finally, environmental identity was positively related with climate change perceptions (*r* = 0.32) and the number of children participants had (*r* = 0.06).

### 3.2 Social psychological characteristics, climate change anxiety and pro-environmental behavior

To explore the role of social psychological characteristics on climate change anxiety and pro-environmental behavior a set of multiple regression analyses was conducted. The results of the multiple regression analyses (Table 3) indicate that parental identity centrality was a significant predictor (β = −0.12, *p* < 0.001), meaning the more central parental identity was, the lower level of climate change anxiety parents experienced.

**Table 3:** Multiple regression analyses predicting climate change anxiety and pro-environmental behavior from identities, climate change perceptions, number of children and age of youngest child.

**Model**	**Climate change anxiety**					**Pro-environmental behavior**				
	**1**	**2**	**3**	**4**	**5**	**1**	**2**	**3**	**4**	**5**
Parental identity centrality	−0.12^***^	−0.12^***^	−0.13^***^	−0.13^***^	−0.13^***^	0.07^**^	0.07^**^	0.06^**^	0.06^*^	0.06^**^
Environmental identity	–	0.23^***^	0.22^***^	0.22^***^	0.22^***^	–	0.36^***^	0.32^***^	0.32^***^	0.32^***^
Climate change perceptions	–	–	0.05^*^	0.05^*^	0.05^*^	–	–	0.15^***^	0.15^***^	0.15^***^
Number of children	–	–	–	−0.01	−0.01	–	–	–	0.01	−0.01
Age of youngest child	–	–	–	–	−0.05^*^	–	–	–	–	0.10^***^
*R^2^*	0.02^***^	0.07^***^	0.07^***^	0.07^***^	0.07^***^	0.01^**^	0.14^***^	0.15^***^	0.15^***^	0.16^***^
*F*_(5, 1998)_	32.312^***^					71.68^***^				

Standardized beta coefficients are reported. Tests of significance were two-tailed. ^*^p < 0.05; ^**^p < 0.01; ^***^p < 0.001.

The results in Table 3 also show that regression equations of parents' environmental identity, climate change perceptions and age of youngest child were all significant. The higher levels of environmental identity participants had, the more they perceived climate change to be real and have negative consequences, the more they experienced climate change anxiety (β = 0.23, *p* < 0.001 and β = 0.05, *p* < 0.05, respectively). On the other hand, the lower the age of their youngest child was, the greater climate change anxiety they expressed (β = −0.05, *p* < 0.05).

Table 3 indicates that parental identity centrality and environmental identity were significant predictors of pro-environmental behavior (β = 0.07, *p* < 0.01; β = 0.36, *p* < 0.001, respectively). The more central parental and environmental identity were the more participants reported pro-environmental behaviors.

Climate change perceptions was also a significant predictor of pro-environmental behavior (β = 0.15, *p* < 0.001). The more participants believed climate change was real, caused by humans and had negative consequences, the higher levels of pro-environmental behavior they declared. Finally, age of youngest child was also a predictor of pro-environmental behavior (β = 0.10, *p* < 0.001), meaning the older their youngest child was, the more pro-environmental behavior participants recorded.

To explore the mediating role of climate change anxiety on the relationship between identities and pro-environmental behavior, methods developed by Preacher and Hayes were followed (Preacher and Hayes, 2004). These analyses were conducted using the PROCESS program (Model 4; Hayes, 2013) with bias-corrected bootstrap estimates and 95% confidence intervals. Table 4 illustrates the results of the mediation analyses. These results indicate that parental identity centrality had an indirect effect on pro-environmental behavior. This effect was mediated by climate change anxiety, as indicated by bootstrap confidence intervals entirely below zero (95% CI [−0.001, −0.001]). On the other hand, the relationship between environmental identity and pro-environmental behavior was not mediated by climate anxiety.

**Table 4:** Bias-corrected bootstrap estimates for parental identity centrality and environmental identity with mediation by climate anxiety.

	**Pro-environmental behavior**		
	**Estimate**	**95% CI**	
		**Lower**	**Upper**
**Parental identity centrality**			
Direct effect	0.003^***^	0.001	0.004
Indirect effect	−0.001^***^	−0.001	−0.001
**Environmental identity**			
Direct effect	0.24^***^	0.207	0.264
Indirect effect	−0.001	−0.006	0.006

CI, confidence interval. ^***^p < 0.001.

## 4 Discussion

This study examined the role of social psychological characteristics on climate change anxiety and pro-environmental behavior. It offers a distinct approach to prior literature by focusing on complex social psychological mechanisms, explaining climate anxiety and pro-environmental behavior beyond demographic characteristics.

The findings revealed that more central parental identities negatively relate and predict climate change anxiety. This extends to previous research on parental identity and mental health (e.g., Fadjukoff et al., 2016; Piotrowski et al., 2024; Schrooyen et al., 2021), revealing that a central parental identity can be a protective factor against mental health issues, including climate anxiety. It further advances our understanding of parental identities and contributes new insights to the experience of climate anxiety (e.g., Clayton and Karzsia, 2020; Patrick et al., 2023). Furthermore, previous research has shown that climate concerns and anxiety are related to young adults' decisions on childbearing, specifically their reluctance and unwillingness to have children (e.g., Diffey et al., 2022; Zimmermann et al., 2024). Therefore, the results highlight the importance of developing a central parental identity and considering becoming a parent as a good decision to prevent mental health issues, including climate change anxiety. As young adults appear to be more ambivalent in their decision to have children, if they do not consolidate such decision and identity, they might be more at risk of suffering from mental health related illnesses when becoming parents.

Parental identity centrality was also found to relate and predict greater engagement in pro-environmental behavior. This can be explained by parents' sense of responsibility toward their children's future, as previous research has shown that higher levels of responsibility to future generations is related to pro-environmentalism (e.g., Syropoulos and Markowitz, 2024).

Environmental identity related and predicted higher levels of climate change anxiety and pro-environmental behavior, replicating and extending to findings of previous studies (e.g., Brügger et al., 2021; Clayton and Karzsia, 2020; Galway et al., 2021) and providing evidence of the role of psychological connection between oneself and the nonhuman natural environment in a representative Portuguese sample.

Additionally, climate change perceptions were also a significant predictor of pro-environmental behavior, echoing and extending to previous findings (e.g., Brink and Wamsler, 2019; Steg, 2023; van Valkengoed et al., 2021) by supplying confirmation for the role of climate change perceptions on conservation behaviors in particular. Taken together these results lead to a better understanding of identities and perceptions of climate change and their impact on pro-environmental behavior, suggesting they can potentially be leveraged to drive conservation behaviors.

Finally, parental identity centrality relates to greater pro-environmental behavior through climate change anxiety, shedding light on the underlying mechanisms that shape more pro-environmental behavior.

The current study strengthens the accumulating evidence of the relevant role played by social psychological characteristics that shape climate change anxiety and pro-environmental behavior (Clayton and Karzsia, 2020; Galway et al., 2021). Mental health and educational professionals should guide parents to appropriate resources and support them to develop adequate skills to tackle, discuss and behave toward climate change anxiety. Additionally, parents should be made aware of the influence that their values, attitudes and behaviors toward the environment has on their children and their experience of climate change. Training should be provided so parents can learn how to foster and sustain a family environment that effectively deals with climate change anxiety and leverage social-psychological characteristics (e.g., parental and environmental identity) to enhance pro-environmental behavior. Training could be incorporated in existing programmes in municipalities/civil parishes (e.g., Eco-Parish programme) or schools (e.g., Eco-Schools programme) as they already funding and staff (mental health and education) to deliver environmental literacy. This is particularly relevant for countries like Portugal, as they face great vulnerability to climate change.

## 5 Limitations and future research

Some limitations of this study should be acknowledged. First, the reliance on self-report measures represents a methodological issue as single-source self-reports could be affected by social desirability and are thus less reliable than a combination of multiple sources of data. Future research would benefit from integrating diverse measurement methods, including experimental designs to assess climate change anxiety and pro-environmental behavior. The choice to use Clayton and Karzsia's (2020) *Climate Anxiety-Scale* introduces a conceptual limitation to the study. The instrument focuses on impairment which has been considered by scholars only one facet of climate anxiety, not measuring other relevant elements such as appraisals and affect (e.g., van Dijk et al., 2025; Wullenkord et al., 2024). The findings should, therefore, be considered with caution and future research would benefit from taking a more holistic approach to climate anxiety and assess all its facets to gain a better understanding of the relationship between social psychological characteristics, climate change anxiety and pro-environmental behavior.

The effect sizes in the study results are quite small suggesting that they should be interpreted with caution. However, they can be recognized as normal in research on complex psychological processes and should not be dismissed (Götz et al., 2022).

Another limitation is the cross-sectional nature of this study, which prevents the extraction of causal conclusions with confidence. More research is needed to establish the causal relationships between social psychological characteristics (namely parental and environmental identities and climate change perceptions), climate change anxiety and pro-environmental behavior, as well as the underlying mechanisms in these relationships. Future studies would benefit from an extensive longitudinal examination of mediating and moderating factors in the relationship between identities, climate change anxiety and pro-environmental behavior.

## 6 Conclusions

This study sheds light on the under-researched role of social psychological characteristics on climate change anxiety and pro-environmental behavior. The results assert the link between parental and environmental identity, and climate change perceptions in experiencing climate change anxiety and motivating people's pro-environmental tendency.

Despite more research being needed to explore potential mediators and moderators of these effects, the current findings provide new insight into the importance of social psychological characteristics and outline potential ways to promote greater mental health and sustainable behavior.

## Data Availability

The raw data supporting the conclusions of this article will be made available by the authors, without undue reservation.
